# Host Genetic Factors Predisposing to HIV-Associated Neurocognitive Disorder

**DOI:** 10.1007/s11904-014-0222-z

**Published:** 2014-07-05

**Authors:** Asha R. Kallianpur, Andrew J. Levine

**Affiliations:** 1 Genomic Medicine Institute, Lerner Research Institute, Cleveland Clinic Foundation, 9500 Euclid Avenue/Mail Code NE50, Cleveland, OH 44195 USA; 2 Department of Molecular Medicine, Cleveland Clinic Lerner College of Medicine of Case Western Reserve University, Cleveland, OH USA; 3 Department of Neurology, David Geffen School of Medicine, University of California, Los Angeles, CA USA

**Keywords:** Combination antiretroviral therapy (cART), Central nervous system, CNS, HIV, HIV-associated neurocognitive disorders, HAND

## Abstract

The success of combination antiretroviral therapy (cART) in transforming the lives of HIV-infected individuals with access to these drugs is tempered by the increasing threat of HIV-associated neurocognitive disorders (HAND) to their overall health and quality of life. Intensive investigations over the past two decades have underscored the role of host immune responses, inflammation, and monocyte-derived macrophages in HAND, but the precise pathogenic mechanisms underlying HAND remain only partially delineated. Complicating research efforts and therapeutic drug development are the sheer complexity of HAND phenotypes, diagnostic imprecision, and the growing intersection of chronic immune activation with aging-related comorbidities. Yet, genetic studies still offer a powerful means of advancing individualized care for HIV-infected individuals at risk. There is an urgent need for 1) longitudinal studies using consistent phenotypic definitions of HAND in HIV-infected subpopulations at very high risk of being adversely impacted, such as children, 2) tissue studies that correlate neuropathological changes in multiple brain regions with genomic markers in affected individuals and with changes at the RNA, epigenomic, and/or protein levels, and 3) genetic association studies using more sensitive subphenotypes of HAND. The NIH Brain Initiative and Human Connectome Project, coupled with rapidly evolving systems biology and machine learning approaches for analyzing high-throughput genetic, transcriptomic and epigenetic data, hold promise for identifying actionable biological processes and gene networks that underlie HAND. This review summarizes the current state of understanding of host genetic factors predisposing to HAND in light of past challenges and suggests some priorities for future research to advance the understanding and clinical management of HAND in the cART era.

## Introduction

Modern combination antiretroviral therapy (cART) has transformed the landscape of clinical complications associated with chronic human immunodeficiency virus (HIV) infection, particularly those involving the central nervous system (CNS). Severe and relentlessly progressive forms of HIV-associated dementia (HAD), linked in the pre-cART era to neuropathological findings such as HIV encephalitis and microglial nodules, are now rare, as are opportunistic CNS infections [1•, 2•, 3]. However, milder forms of HIV-associated neurocognitive disorder (HAND) such as asymptomatic neurocognitive disorder (ANI) and mild neurocognitive disorder (MND) [4] are increasingly prevalent, owing to the intersection of chronic immune activation, effects of aging, and antiretroviral drug toxicities [5•, 6•, 7–9, 10•]. HAND is diagnosed in 40-50 % of unselected, chronically HIV-infected individuals in the cART era who undergo formal neuropsychometric testing [1•, 11]. In contrast to HAND in the pre-cART era, cortical brain involvement, including impairment of learning, memory, and executive functions, often predominates over impairment of subcortical and motor functions in patients on cART, similar to non-HIV-associated neurocognitive disorders like Alzheimer’s dementia [1•]. While not as devastating as HAD, milder neurocognitive impairment (NCI) adversely impacts medication adherence (particularly in older HIV-infected persons), performance of cognitively demanding activities of daily living [1•, 12], employment, and overall quality of life [5•]. Increased risky decision-making and shorter survival among HIV-infected persons with HAND are also well documented [13•, 14, 15].

The neuropathogenesis of HAND remains incompletely understood; it may overlap that of other common neurodegenerative diseases in which genetics has a role and with which HAND shares certain similarities [16, 17•, 18•, 19, 20•]. Recognition of the critical importance of neuro-inflammation, reflected by elevated expression of inflammation or immune-activation biomarkers in the brain, cerebrospinal fluid (CSF) and in some cases, plasma in HAND [21–23], and the central role played by mononuclear phagocytes in these disorders, has provided a framework for studies of the role of host genetic variation in HAND to date [18•, 24•, 25•].

The purpose of this review is to summarize the current state of understanding of host genomic factors that predispose to HAND with an emphasis on recent studies and reasonable conclusions that may be drawn from this rapidly growing volume of data. We conclude with a discussion of some research priorities and suggestions for a way forward in this complex field.

## Host Genomic Studies

Host genetic variation is likely to impact both adaptive and maladaptive host responses to HIV infection that play a role in neuropathogenesis, just as host genetics clearly impacts susceptibility to HIV infection and the rate of disease progression [26–30]. Many candidate-gene studies and a single genome-wide association study spanning the pre-cART and cART eras, primarily focusing on HAD with or without HIV encephalitis, have identified variants in immune-regulatory genes and other gene classes as potential risk-modifiers or protective factors, but very few of these genes have been replicated in subsequent studies (Table 1) [31•, 32•]. A number of factors that complicate the clinical definition, risk-stratification, and monitoring of HAND, have presented challenges to genetic studies aimed at better understanding susceptibility to these disorders: inherent fluctuations in individual neuropsychometric test scores over time, despite correction for practice effects, imprecision of diagnostic categories like ANI and MND, possible residual biases in testing protocols, and varying methods used to determine composite scores in prior published studies of NCI among HIV + persons. Distinguishing reversible NCI from the “legacy effect” of brain damage due to previously untreated HIV infection, and completely excluding confounding by comorbidities also remain difficult [6•, 7]. In contrast to the pre-cART era, many converging pathogenic mechanisms are likely to contribute to HAND at present [33, 34, 35•]. These challenges notwithstanding, recent genomic, transcriptomic, and epigenomic studies have highlighted metabolic pathways and physiologic processes that are disrupted in HAND (Table 2).

**Table 1:** Summary of published genetic associations (positive findings) in candidate-gene and genome-wide association studies (GWAS) of HIV-associated neurocognitive disorder (HAND) (*See text for references*). Only genes associated with a HAND-related phenotype in at least one published study, and studies that included HIV + subjects, are listed

Genes/processes dysregulated in HAND	Clinical phenotype(s) evaluated^1^	Study design(s)	Replication status^2^
*Nuclear genes*			
*APOE* (*E4 allele*)	AIDS with ADC/HAD ± HIVE; non-AIDS with HAND ± neuropathologic features	Autopsy (mostly case-control; one survival study with autopsy component; 2 uncontrolled); cross-sectional; longitudinal cohort	R
*TNFA*	HAD; HAD/ADC, or HIVE and/or HIV-LE	Autopsy case-control	NR
*MCP1*/*CCL2*, *CCR2*	HAD ± HIVE or AIDS/ADC, *OR* change in executive functioning and processing speed between 2 consecutive visits up to 15 yrs apart; or NCI (clinical rating score ≥ 5); HAE (children)	Retrospective case-control; longitudinal cohort ± cross-sectional analysis	R (*MCP1*) NA (*CCR2*)
*MIP1A*/*CCL3*	HAD; AIDS with HAD; *OR* change in executive functioning and processing speed between 2 consecutive visits up to 15 yrs apart; *OR* risk of NCI	Retrospective case-control; longitudinal cohort	R
*SDF1*	Decline in NC test scores and/or brain growth failure in children; *OR* change in executive functioning and processing speed between 2 consecutive visits up to 15 yrs apart; *OR* prevalent NCI (adults); change in GDS or cross-sectionaL GDS in co-HCV+	Longitudinal cohort with cross-sectional component; retrospective case-control	NR
*MBL2*	Changes in GDS or cross-sectional GDS in co-HCV+; *OR* change in executive functioning and processing speed between 2 consecutive visits up to 15 yrs apart; *OR* prevalent NCI (adults)	Longitudinal cohort with cross-sectional component	NR
*CCR5* (*δ32 del*)	HAD/ADC; AIDS ± HAD; decline in NC test scores and/or brain growth failure in children; NCI in children; GDS (change and cross-sectional)	Longitudinal cohort ± cross-sectional component; case-control	R prior to 1991 only; NR in cART era
*COMT*	Executive functioning domain Deficit Scores ± stimulant abuse; HAND: standardized NP domain T-scores	Retrospective/Case-control	NR
*DRD2*, *DRD3*	GDS ≥ 0.5 (NCI); Global and cognitive domain T-scores in population with prevalent substance dependence	Cross-sectional/Case-control	R (DRD3 in substance users)
*HLA*:*DR*, *DQB1*, *A24*, *B27*	Time to CNS impairment (“deterioration in brain growth, psychological function and/or neurological status”)	Pre-cART cross-sectional study; cART era case-cohort study; longitudinal cohort	R (*DR*, *B27*) NA (*DQB*) NR (*HLA A*)
*APOBEC3G*	Brain growth failure, with NCI defined differently based on age	Pre-cART pediatric cohort study	NA
*PKNOX1*/*PREP1*	AIDS with dementia	Retrospective case-control	NA
*YWHAE*	HAND	Cross-sectional study with HIV+/HIV- controls	NA
*Mitochondrial & nuclear DNA structural changes*			
8-oxoG modification	HAND “screen”, International HIV Dementia Score ≤ 10	Autopsy case-control	NA
Regulation of telomere length	Detailed NP test scores (global and ability domain scores) ± history of chronic psychological trauma (Childhood Trauma Questionnaire Short Form)	Cross-sectional with HIV+/HIV- controls	NA

^1^Diagnostic criteria used included one or a combination of the following: American Academy of Neurology 1991 criteria, Centers for Disease Control criteria, Frascati criteria, the Global Deficit Score (GDS), Domain Z-scores or Global Z-scores, the HIV Dementia Scale, neurocognitive impairment >1.5 SD below the normative mean in two domains on comprehensive test battery, Diagnostic and Statistical Manual of Mental Disorders (DSM) III criteria for dementia, or unspecified.

^2^Replication status: R = Replicated in at least one other candidate-gene study; NR = Did not replicate in at least one study; NA = No published replication attempts. Importantly, no genes/SNPs previously associated with HAND replicated in the GWAS.

*Abbreviations*: *ADC*, AIDS dementia complex; *HAD*, HIV-associated dementia; *HIVE*, HIV encephalitis; *HIV*-*LE*, HIV leukoencephalopathy; *HAND*, HIV-associated neurocognitive disorder; *HAE*, HIV-associated encephalopathy; *NP*, neuropsychiatric; *NC*, neurocognitive; *NCI*, neurocognitive impairment; *GDS*, global deficit score; *HCV*, hepatitis C-virus. *Gene names*: *APOE*, apolipoprotein E; *TNFA*, tumor-necrosis factor-alpha; *MCP1*/*CCL2*, macrophage chemoattractant protein-1; *CCR2*, C-C chemokine receptor type 2; *MIP1A*/*CCL3*, macrophage inhibitory protein-1-alpha; *SDF1*, stromal derived factor 1; *CCR5*, C-C chemokine receptor type 5; *del*, deletion; *MBL2*, mannose-binding lectin 2 ; *COMT*, catechol-*O*-methyltransferase; *DRD*, dopamine receptor; *CYP2D6*, cytochrome P450 2D6; *CYP2B6*, cytochrome P450 2B6; *HLA*, human leukocyte antigen locus; *APOBEC3G*, apolipoprotein B mRNA-editing, enzyme-catalytic, polypeptide-like 3G; *PKNOX1*/*PREP1*, PBX knotted 1 homeobox 1; *YWHAE*, 14-3-3ε protein (Tyrosine 3-monooxygenase/Tryptophan 5-monooxygenase activation protein, epsilon isoform)

**Table 2:** Genes with significantly altered expression in transcriptomic and epigenetic studies and hence implicated in HAND pathogenesis

Gene categories and pathways dysregulated in HAND-related phenotypes	Source material/ phenotype(s)	Reference(s)
*Monocytes*		
* Pathways*:	Brain metabolite neuroimaging traits, *e.g*., *N*-acetylaspartate concentration in FWM, anterior cingulate	Pulliam et al. 2011 [100]; Borjabad, 2012 [17•]
IFN response/activation		
* Example genes*: *IP*-*10*		
* Pathways*:	Neurocognitive impairment (GCR)	Levine et al. 2014 [31•]
Mitotic cell cycle		
Translational elongation		
Oxidative stress		
* Example genes*: *IL6R*, casein kinase 1-alpha-1, hypoxia upregulated-1, LDL receptor-related protein 12, *KEAP*-*1*		
*Brain tissue*/*brain*-*derived cells*		
* Pathways*:	SIV ± SIVE, HIV ± HIVE brains; also primary neurons *in vitro*	Eletto et al. 2008 [104]; Yelamanchili et al. 2010 [106]
pre-synaptic proteins/general neuronal function		
* Example genes*: miR-128a, *SNAP25*, *MEF2C*, miR-142-3p, miR-142-5p, miR-21 (downregulation of target *MEF2C* in neurons)		
* Pathways*:	FWM from HIV-, HIV+/HIVE brains; immunohistochemistry; glutamate levels, brain neurotrophic factor levels	Noorbakhsh et al. 2010 [107]
IFN response		
Neuroinflammation	Gene expression in primary astrocytes exposed to HIV Vpr protein *in vitro vs*. controls	
Nucleotide metabolism		
Cell cycle	Neurobehavioral abnormalities (locomotion) in transgenic mice with increased BG Vpr expression	
Mitochondrial function		
Apoptosis (astrocytes)		
* Example genes*: *Caspase*-*6*		
* Pathways*:	Acute and chronic SIV model ± SIVE	Reviewed in Winkler et al. 2012 [92•]
Inflammation		
Immune and acute-phase response		
Ion transport		
* Example genes*: *B2M*, *STAT1*, *IFI44*, *IFIT3*, *MX1*		
* Pathways*:	HAND diagnosis (±cART), ±HIVE	Borjabad et al. 2012 [17•]
Neurogenesis		
Synaptic transmission		
Antiviral/immune responses (including IFN responses, complement activation)		
Ion/Calcium transport		
Antigen presentation/processing		
Oxygen transport		
Signal transduction		
Cell cycle		
Oligodendrocytes/Myelination		
Microtubule-based movement		
* Example genes* (*treated vs untreated HAND*): *CXCR2*, *CR1*, *HLA*-*DQB1*, *CXCL2*, *IFIT1*, *IFI44*, *STAT1*, *MOBP*		
* Pathways*:	HAND ± HIVE *vs.* HIV+/HAND- *vs.* HIV- controls; neurocognitive impairment (GCR)	Gelman et al. 2012 [93•]; Levine et al. 2013 [94•]
Synaptic transmission		
Neuroimmune function		
Endothelial markers		Gelman et al. 2012 [93•]; Levine et al. 2013 [94•]
Neuronal function		
Glutamate signaling		
Axon guidance		
Clathrin-mediated endocytosis		
IFN response		
Antigen presentation/processing		
Inflammation/acute-phase response/toll-like receptors		
Oligodendrocyte function		
Mitochondrial function		
Cell signaling		
Protein ubiquitination		
Caveolin-mediated endocytosis		
* Example genes*: *YWHAE*/14-3-3 protein, *GAD1*, *IFRGs*, *TFRC*		
* Pathways common between HAND and AD*:	HAND ± HIVE brains *vs.* HIV+/HAND-, HIV+/HAND-; neurocognitive impairment (GCR) in HIV + *vs*. Mini-Mental Status Exam data in AD	Levine et al. 2013 [94•]
Cytoplasm		
Mitochondrial function		
Tricarboxylic acid cycle		
Transit peptide		
Synaptic		
Cell Differentiation		
Activator		
Repeat		
Cell communication		
Regulation of transcription		
Phosphorylation		
* Pathways differentiating HAND* + *vs.* – *HIVE*:		Levine et al. 2013 [94•]
Gliosis		
Dopaminergic tone Inflammation		
* Example genes*: *GRK6*, *CCL2*, *ID2*		
* Pathways*:	HAND *vs*. HIV- controls	Lucas et al. 2014 [113•]
Neuronal RNA splicing/gene transcription		
* Example genes*: *RbFOX3*		
* Pathway*:	Global and cognitive domain T-scores/GDS (all 7 domains)	Jacobs, 2013 [72•]; Gupta, 2011 [71]
Dopaminergic response		
* Example genes*: *DRD3*		
* Pathways*:	Subsyndromic HAND/ANI, MND or HAD ± HIVE, *vs*. HIV- controls	Desplats et al. 2013 [117•]
Chromatin modification		
Inflammation		
* Example genes*: *BCLB11B* (targets *IL*-*6*, *TNFα*, *CXCR4*)		

*Note*: Studies that lacked correlation with at least one *in vivo* HAND-related phenotype are not listed.

*Abbreviations*: *miRNA*, microRNA; *SIV*/*E*, simian immunodeficiency virus/encephalitis; *HIV*/*E*, human immunodeficiency virus/encephalitis; *FWM*, frontal white matter; *HAND*, HIV-associated neurocognitive disorder; *GCR*, Global Clinical Rating; *GDS*, Global Deficit Scores; *BG*, Basal Ganglia; *AD*, Alzheimer’s Disease

### Candidate-Gene and Genome-Wide Association Studies

#### Genes related to inflammation or immune regulation

Genes that have been inconsistently linked to HIV-associated neurocognitive phenotypes, including HAD with or without HIV encephalitis, include: apolipoprotein E (*APOE*) [29, 36•, 37•, 38, 39•, 40–45], tumor necrosis factor-α (*TNF*-α) [31•, 32•, 42, 46–48, 49•, 50, 51], macrophage chemo-attractant protein-1 (*MCP*-*1*/*CCL2*) [21, 22, 32•, 48, 49•, 52–56, 57•], macrophage inflammatory protein 1-α (*MIP*-*1*α/*CCL3*) [32•, 48, 49•, 57•], stromal cell-derived factor-1 (*SDF1*) [31•, 49•, 56, 58], *HLA* alleles [59, 60•, 61•], mannose-binding lectin 2 (*MBL2*) [49•, 56, 57•, 62, 63], and *PKNOX1*/*PREP1*, a transcriptional regulator of *MCP*-*1* expression [32•, 49•]. Associations of C-C chemokine receptor 2 (*CCR2*), the co-receptor for MCP-1, with HAD or AIDS dementia complex, progression to NCI in adults, or NCI among children have been similarly inconsistent [32•, 54, 56, 57•, 58, 64]. While the Δ32 deletion variant of C-C chemokine receptor type 5 (*CCR5*) [65] was protective against HAND in pre-cART populations [58, 64, 66], this association has not been replicated in individuals with HIV/AIDS diagnosed after 1991, possibly due to reduced impact on viral load in the cART era [32•, 54, 56]. Preliminary results from a recent cross-sectional study among CNS HIV Antiretroviral Therapy Effects Research (CHARTER) study subjects suggest that a polymorphism in the *MBL2* promoter (rs7096206) may predict altered levels of brain metabolites in frontal white matter and basal ganglia, reflecting increased neuro-inflammation and energy dysmetabolism in the brain [67]. In the largest longitudinal study to date, drawing from pre-cART neurocognitive data from HIV + and seronegative participants in the Multicenter AIDS Cohort Study (MACS), Levine et al. [57•] did find evidence for a small effect of *MIP*-*1α*/*CCL3* and *MCP*-*1*/*CCL2* genotype on neurocognitive functioning over time among HIV + cases only, but the magnitude of this effect was small.

Interaction effects between *MCP*-*1* and SNP rs2839619 in the *PREP1* locus, which encodes a transcription factor that binds and activates the *MCP*-*1* promoter, may explain inconsistencies in *MCP*-*1* associations with HAND. *PREP1* genotype (rs2839619) has been associated recently with HAD (*p* = 1.2 x 10^-5^), and there is extensive support for a role for MCP-1 protein in the etiology of HAD in particular [32•]. *PREP1* did not replicate in the single published GWAS of HAND (largely HAD), however [49•].

#### Dopamine-related genes

Some genes involved in dopamine pathways have been also been variably associated with neurocognitive function in HIV + persons. The Met/Met genotype at the catechol-*O*-methyltransferase (*COMT*) locus (rs4680) predicted improved executive functioning in HIV + subjects, an association that was attenuated by methamphetamine (meth) use [68]. No main effects on HAND, or interactive effects of *COMT*, dopamine transporter-1 (*DAT1*), or brain-derived neurotrophic factor (*BDNF*) with other measures of HIV disease severity (*e.g*., CD4+ T-cell count), were noted on specific domains of neurocognitive function in HIV-infected individuals in other studies, including the GWAS [49•, 69•]. The recent longitudinal study in the MACS did not detect any interactions between stimulant use, HIV status or dopamine-related genes (*COMT*, *BDNF*, dopamine beta-hydroxylase, and *DRD2*/*ANKK1* genotypes) on neurocognitive functioning in HIV + subjects [57•]. Neurotrophin genotypes may assume greater importance in HAND among older individuals [70]. The dopamine receptor gene *DRD3* (rs6280) was associated with NCI only among HIV + meth abusers [71], but significant main and interaction effects with substance use have also been seen for dopamine receptors 1 and 2 (*DRD2*) in the motor domain of neuropsychological performance among whites, and with nearly every neuropsychological domain among African-American subjects [72•]. The GWAS conducted in the longitudinal MACS found null associations with *DRD2* or *DRD3* but did not address interactions with substance/stimulant abuse.

#### Drug metabolism and transport genes

Genetic differences in meth metabolism have been proposed to be a factor in individual variation in risk of NCI among HIV-infected persons who abuse meth. Oxidative metabolism of meth requires the hepatic enzyme cytochrome P450, family 2, sub-family D, polypeptide 6 (CYP2D6). Extensive metabolizers based on polymorphisms in *CYP2D6* were significantly more vulnerable to meth-associated NCI in a recent study that included no HIV + subjects, suggesting that *CYP2D6* genotype might also be a risk factor for HAND among HIV + meth abusers [73].

Suppression of HIV replication in the CNS is a key component of any strategy to reduce risk of HAND, and there remains some evidence that better-penetrating antiretroviral regimens are helpful in preserving neurocognitive function [74, 75•]. Highly active antiretroviral drugs vary considerably in their ability to penetrate the blood-brain barrier (BBB). Recent pharmacogenetic/pharmacokinetic studies were unable, however, to associate the common variant 3435C > T (rs1045642) in the drug efflux transporter gene, ATP-binding cassette transporter P-glycoprotein *ABCB1*, or 150 other polymorphisms in 16 membrane transporter genes, with CSF raltegravir levels in healthy HIV-negative subjects [76•]. As noted by the authors, this study was underpowered to detect associations for SNPs in many genes with low minor-allele frequencies in the sample. A similar study, also in seronegative individuals, evaluated the pharmacokinetics of efavirenz, which is metabolized by hepatic CYP2B6 [77] and associated with CNS toxicity, following a single oral dose (600 mg) of the drug. Polymorphic variants of *CYP2B6* (516G > T, rs3745274) and 983 T > C (rs28399499), which classify subjects as, extensive, intermediate, or slow metabolizers and are known to impact efavirenz plasma area-under-the-curve, were not significantly associated with neurocognitive performance, although *CYP2B6* genotype tended to correlate with non-dominant hand grooved pegboard at 4 and 6 hours after the dose [78•]. Since the CNS side effects of this drug generally wane with repeated use, it is unclear to what extent *CYP2B6* genotype may impact NCI in treated subjects.

#### Nuclear and mitochondrial DNA damage

The role of oxidative damage to nuclear and mitochondrial DNA (mtDNA), which can lead to neuronal apoptosis, in the pathogenesis of milder forms of HAND seen in the cART era is not clear. Neurotoxicity of HIV proteins such as Tat, gp120, and Vpr in the cART era is mediated by excitotoxic neurotoxins released by infected microglia and macrophages, activation of intracellular and mitochondrial calcium signaling pathways, and increased production of reactive oxygen species [79•]. One study compared levels of nuclear and mtDNA oxidative damage (8-oxoG modification) in postmortem frontal neocortex tissue of HIV + patients with AIDS with and without HAND and in seronegative controls. Nuclear DNA 8-oxoG damage was significantly higher in HIV + patients, particularly those with HAND, and a substantially higher frequency of noncoding D-loop mutations in mtDNA among HAND cases was noted as compared with either HIV + or seronegative controls. Possible confounding by age was not mentioned [79•, 80]. These results leave open the possibility that as long as a reservoir of virus exists in the brain, oxidative damage may continue to contribute to the development of HAND.

A more recent study of South African women examined the association of leukocyte telomere length (TL) with HAND and chronic psychological stress due to prior abuse and/or trauma [81•]. Telomeres are repetitive nucleotide sequences at the ends of chromosomes that shorten by 20-200 base-pairs with each mitotic division, due to the inability of DNA polymerase to complete DNA replication at the ends of each strand. Activity of the enzyme telomerase, which synthesizes telomeric DNA to prevent excessive shortening, is reduced by oxidative stress, and shorter TL has been reported under chronic psychological stress conditions. In analyses adjusted for age and education, TL in HIV + women was found to be significantly shorter relative to controls and was strongly correlated to severity of NCI, as determined by the International Neuropsychological Battery. A negative correlation of relative TL was also found with verbal fluency in HIV + women who had suffered psychological trauma. This was the first study to investigate the effects of TL on neurocognitive function in HIV-positive individuals. Very early but intriguing results of a retrospective study presented at the 2014 Conference on Retroviruses and Opportunistic Infections [82] reported strong positive associations between levels of cell-free mtDNA in CSF, another potential novel marker of neuronal injury, and severity of NCI among HIV-infected persons with HAND, as well as with biomarkers of CNS inflammation and immune activation (MCP-1 and interferon-gamma-inducible protein (IP-10), respectively).

Miscellaneous genes that have been linked in published reports to HIV-associated neurocognitive phenotypes, but for which studies attempting replication have not been published, include *HLA DQ* [60•] and *APOBEC3G* (in pre-cART pediatric populations) [83•], and YWHAE (among Hispanic-Latino populations) [84•, 85]. Several genes associated previously with HIV replication in monocyte-macrophages or with neurotoxic protein production *in vitro* have not been associated with HAND [32•, 86, 87•].

### Transcriptomic and Epigenetic Studies

The advent of microarray technology has led to numerous studies investigating the effects of HIV infection on host gene expression in phenotypes that contribute to but are relatively distant from HAND, including viral replication, HIV persistence, apoptosis, and immune dysregulation in general. Those studies performed prior to 2007, have been reviewed extensively elsewhere [88] Subsequent functional genomics studies have yielded additional information on genes and pathways up- or down regulated in astrocytes, neurons, and glial cells, cell types intimately involved in HAND pathogenesis (Table 2) [31•, 89–91]. A number of genes show consistently altered expression across studies of human astrocytes *in vitro*, the human HIV-infected brain with or without HAD or HIVE, and the simian-immunodeficiency virus (SIV)-infected macaque model, pointing to their likely importance in HAND [31•, 90, 92•]. While many studies to date have focused on patterns of RNA expression changes specific to HIVE in frontal gray matter [92•], few have investigated differences in brain regions affected by HAND without HIVE. A microarray study by Gelman et al. [93•] using brain tissues from HIV-infected and seronegative individuals enrolled in the National NeuroAIDS Tissue Consortium (NNTC) Brain Bank, revealed two different transcriptome patterns in HAND + HIVE and HAND alone. HAND combined with HIVE was associated with high viral load, global upregulation of genes involved in interferon responses, and general immune activation, while specific neuronal transcripts in frontal neocortex were down-modulated. HAND without HIVE, however, was associated with low viral load, upregulated endothelial-type transcripts, and the conspicuous absence of gene-expression changes noted in HIVE. Weighted-gene coexpression network analysis (WGCNA) of the same transcriptomic data, accounting for correlations amongst functionally related genes, also identified meta-networks of genes associated with global neurocognitive function; these included cancer-related genes and genes important in oligodendrocyte function [94•] in frontal neocortex, frontal white matter, and the basal ganglia. Dysregulation of genes involved in mitochondrial function, cancer, the immune response, synaptic transmission and cell-cell signaling has also been suggested in other studies of HAND [17•, 94•] (Table 2).

The role of antiretroviral drug toxicities in HAND remains controversial. The contribution of complex drug interactions, side effects when taking an increased number of drugs for advanced disease, and known mitochondrial effects of older dideoxynucleoside reverse-transcriptase-inhibitors such as stavudine and didanosine, to NCI is unknown [8, 95]. Transcriptomic studies evaluating the impact of cART on gene expression patterns in HAND in brain tissue reveal alterations in expression of about 100 immune-regulatory, cell-cycle, and myelin-pathway genes that are not correlated either with brain viral burden or to antiretroviral drug CNS penetration effectiveness (CPE) score, suggesting a possible explanation for the difficulty to date in correlating CPE scores to neurocognitive outcomes despite their association with reduced CSF viral load [8, 96•].

HIV-infected monocytes or monocytes from HIV-infected as compared to seronegative individuals have been the focus of many *in vitro* microarray-based studies to identify upstream biological mechanisms relevant to HAND, due to their key role in BBB injury. Genes and pathways that have been found to be significantly upregulated in such studies include: a large number of chemotaxis- and inflammation-related genes [97, 98] and genes involved in the interferon (IFN) response, as well as genes that promote antioxidant and anti-inflammatory responses [31•, 99, 100]. Expression of subsets of these genes have also been associated in some, but not all [99] studies, with mild NCI [31•, 100], with HAND in hepatitis C/HIV-co-infected subjects on cART [101•], and with metabolic neuroimaging traits such as *N*-acetyl-aspartate in frontal white matter [100]; however, monocyte transcriptome patterns have not always correlated with NCI in HIV + persons [99, 102].

A recent postmortem study examined changes in expression of ephrin (*EPH*) genes that mediate synapse formation and recruitment of glutamate receptors to synapses [103•]. Postmortem brain tissues from cognitively characterized HIV-infected subjects and seronegative controls from the Manhattan HIV Brain Bank were examined for levels of expression of a variety of genes, including *EPHA4* and *EFNB2* (an ephrin ligand). Transcript levels of both of these genes in the caudate, and of *EPHB2* in the anterior cingulate were significantly lower in HIV-infected patients, and *EPHB2* mRNA levels in the cingulate correlated with premortem neurocognitive function. The authors hypothesized that decreased expression of *EPHB2* in the cingulate may represent a compensatory mechanism minimizing excitotoxic injury in the face of chronic inflammation.

The small number of published epigenetic studies of HAND have focused on expression of microRNA (miRNA), small non-coding RNA molecules that bind messenger RNA and regulate gene expression at the transcriptional or post-transcriptional levels. MicroRNA (miRNA) expression studies conducted in cortical neurons exposed to viral proteins such as Tat and Vpr [104, 105•], or in tissue from individuals with HIVE or SIV-infected macaques with encephalitis (SIVE) have implicated upregulation of the following classes of host miRNAs in HIVE and SIVE: 1) immune response and inflammation, 2) nucleotide metabolism, 3) cell cycle, 4) oncogenesis (*e.g*., miR-21, which targets a neuronal transcription factor), and 5) apoptosis (*e.g*., caspase-6). Downregulated miRNAs included those involved in: 1) inflammation, 2) neuronal monoamine oxidase activity (possibly explaining the reduced dopaminergic activity in HAND), 3) apoptosis (*e.g*., suppression of caspase-6 expression), 4) modulation of viral replication, 5) mitochondrial function, and 6) axonal guidance (Table 2) [105•, 106–108, 109•, 110•, 111•]. These studies have provided some useful leads and validated several neuropathogenic mechanisms in HAND, but sample sizes have been extremely small (five individuals or less in human studies). In general, these studies have not evaluated associations with neurocognitive phenotypes or accounted for multiple statistical tests or potential confounders in the analyses. Findings in SIV models also require replication in humans.

Consolidation of short-term memories into long-term memory requires synaptic plasticity, which is characterized by structural changes and altered gene expression at neuronal synapses [112•]. In keeping with the finding that synaptodendritic damage rather than neuronal loss is a neuropathological feature of milder forms of HAND [113•], a recent study found downregulation of many synaptic plasticity genes in HIV-infected astrocytes, and increased expression of pro-apoptotic genes, compared to uninfected controls [112•]. These findings translated into reduced dendritic spine density and altered dendritic morphology, which were most prominent in cells infected with clade B virus.

Finally, Lucas et al. [113•] have reported a highly abnormal distribution of the RNA splicing factor NeuN/Rbfox3 in postmortem brain tissue from 22 HIV-infected individuals with MND/HAD as compared to seronegative controls. Very few targets have been identified for this splicing factor, which is usually confined to the nucleus, where RNA splicing occurs. The authors posit that altered localization of RbFox3 in HAND may reflect downregulation of expression of neuronal genes relevant to HAND pathogenesis. This finding requires further study.

Relatively few studies have evaluated the role of histone modification and DNA methylation in the context of HAND. Histone deacetylases (HDACs) function in epigenetic regulation by deacetylating histones and other proteins involved in transcription and chromatin remodeling; histone hypoacetylation has been linked to many neurodegenerative diseases. Saiyed et al. [114•] showed that HIV-1 Tat protein upregulates HDAC2 expression in neuronal cells, leading to transcriptional repression of genes involved in synaptic plasticity and neuronal function and suggesting a potential therapeutic role for HDACs as a drug class in HAND [115, 116]. More recently, Desplats et al. conducted a case-control study among 32 deceased HIV + individuals from the HIV Neurobehavioral Research Center and California NeuroAIDS Tissue Network, 72 % of whom underwent neurocognitive testing within 1 year of death [117•]. The study compared epigenetic markers in postmortem brain tissue, such as B-cell CLL/lymphoma (BCL11B), a transcriptional silencer, among several patient groups: HIV + controls without detectable proviral DNA, RNA or p24 in the CNS, HIV + cases with high viral DNA but no HIV RNA or p24 (latent cases), and HIVE cases with high expression of viral DNA, RNA and p24. Up to half of HIV-infected subjects were on cART, and with the exception of HIVE cases, all HIV + subjects had mild to moderate NCI. Compared to HIV + controls, higher levels of BCL11B protein and other chromatin modifiers involved in transcriptional silencing of HIV-1 (including HDAC1) were observed in HIV + latent cases and were associated with dysregulation of pro-inflammatory genes like *IL6*, *TNFA*, and *CXCR4*. Latent cases also displayed more cognitive impairment than HIV + controls. These results suggest that even in the absence of detectable viral replication, significant dysregulation of pro-inflammatory genes may still occur and that these changes are associated with increased levels of epigenetic factors such as BCL11B. These findings are highly relevant to strategies for eradicating viral reservoirs which might include modulation of BCL11B.

Narasipura et al. [118•] examined the role of epigenetic regulation in maintaining latency of the virus in astrocytes *in vitro*. DNA CpG methylation and histone modifications (methylation and deacetylation) at the HIV-1 promoter region are specific hallmarks of HIV-1 latency, and HDAC inhibitors reactivate the virus in cell culture models and in HIV-infected CD4+ T cells [118•, 119•]. This study added to previous studies by demonstrating the role of epigenetic regulation in maintaining and reversing virus latency in astrocytes specifically, a process implicated in HAND [90]. Inhibitors of class I HDACs and histone methyltransferases which demethylate DNA are able to activate the HIV-1 promoter in latently infected astrocytes, thereby confirming that these cells may be clinically important reservoirs for HIV in the brain. Other *in vitro* studies have revealed epigenetic regulation of markers in T-regulatory cells, which normally maintain gut-mucosal immune tolerance via suppression of effector T-cell functions but are dysregulated in chronic HIV infection [120•], as well as dysregulation of HDAC1 and DNA methyltransferases in oral epithelial cells, potentially contributing to HAND via increased oral microbial disease and peripheral immune activation [121].

We know of only one unpublished study of DNA methylation in the context of HAND, the preliminary results of which were presented at the 19th Conference on Retroviruses and Opportunistic Infections in 2012 [82]. This study of 17 HIV + participants in a longitudinal cohort study showed many strong positive and negative correlations of methylation profiles at autosomal sites with changes in neurocognitive test performance (scaled scores corrected for practice effects) measured at two consecutive time points.

Other very preliminary findings deserving of further exploration and replication in studies of HAND include: 1) interactions between opioid-related genes such as OPRM1, substance abuse, and HAND [122•]; 2) mitochondrial DNA haplogroup effects on HAND risk within specific ethnic subpopulations [123]; 3) the impact of iron metabolism, which is essential for mitochondrial function as well as dopaminergic metabolism [124, 125•]; and 4) potential protection against HAND by promoter variants in the antioxidant response gene HMOX1 [126].

## Summary

Many candidate-gene studies have identified genetic variants as risk or protective factors in HAND, but due to a combination of factors—study heterogeneity, application of different diagnostic methods, low power, changing epidemiology—few of these genes and variants have been reliably replicated; nor have any prior associations been replicated in the single, published genome-wide association study. Due to significant differences in study designs and methodologies, estimates of effect size and measures of significance are not very meaningful even for genes/SNPs that have been replicated at least once. A large number of genes and biological pathways have also been implicated in genome-wide transcriptome and epigenetic studies, some of which are consistently revealed across human, non-human primate (SIV) models, and murine models of HAND. Human studies have largely employed homogenized brain tissue reflecting multiple cell types, however, complicating the dissection of cell-specific processes that are dysregulated. Finally, not all of the genetic and transcriptomic studies in HAND have incorporated adjustments for multiple statistical comparisons, increasing the likelihood of false-positive findings amid true associations. In some cases, however, published and preliminary studies of two or more types— candidate-gene, GWAS, and transcriptomic—have yielded consistent evidence regarding *mechanisms* of HIV neuropathogenesis, as in the case of genes involved in inflammatory or immune regulation, synaptic plasticity and neuronal function, iron transport [*e.g*., transferrin receptor (*TFRC*)], and mitochondrial function [17•, 93•, 94•, 104, 123, 124, 127•, 128].

It is important to remember that HAND is an extremely complex phenotype, influenced by numerous environmental, psychological, lifestyle and endogenous host factors; it is likely to involve alterations in many brain regions that normally compensate for one another. Studies to date have mostly evaluated only changes in the frontal cortex, rather than white matter, hippocampus, and basal ganglia regions that also show abnormalities in HAND [129•, 130, 131•]. The focus has also been on severe HAND phenotypes such as HAD and HIVE, which probably no longer reflect the salient biological mechanisms that contribute to milder forms of HAND today [132]; individuals with HAND and postmortem HIVE have distinctly different transcriptomic profiles from those with HAND alone [93•]. It also seems likely that relatively little risk is attributable to individual genes, and that these small effect sizes are easily obscured by differences between studies and methodologies used. This poses a challenge even for meta-analyses that might be done in a consortium context with larger sample sizes.

Dysregulation of genes involved in synaptic plasticity, axonal guidance, and interferon response is also a relatively consistent theme. Perhaps the most important conclusions to be drawn from the impressive body of data derived from GWAS, candidate-gene, transcriptomic and epigenetic studies thus far are that: 1) *APOE* genotype may play a role in older HIV-infected persons [6•, 133•]; 2) genes involved in inflammation and immune regulation in the periphery and CNS, macrophage/monocyte responses to HIV infection, synaptic plasticity, axonal guidance, and mitochondrial function are fundamental in determining neuronal injury and ultimately, neurocognitive function in HIV + individuals; 3) specific gene modules and molecular pathways revealed to be dysregulated in HIV-infection and HAND may now be evaluated for individual variation and therapeutic potential; and 4) dopaminergic dysfunction is altered in HAND and may play a role in HAND pathogenesis among subsets of HIV-infected substance users [91, 134]. Many of these findings still require replication. The potential impact of host genetics on epigenetic modifications in HAND is also an unexplored area that may improve understanding of maladaptive host responses to HIV infection [135•].

## Future Research Priorities in the Genomics of HAND

### Study populations and design

There is an urgent need for longitudinal studies of HAND in which subjects serve as their own controls, minimizing the impact of confounding factors and optimizing power for detection of genetic effects. There has been a dearth of studies involving pediatric populations, in whom the long-term effects of HIV infection on the CNS may be particularly devastating, and in women, who may have less access to care in some settings and/or may be more vulnerable to the effects of substance dependence on some aspects of neurocognitive function [136–138].

It is currently unclear whether global indices of NCI, even when adjusted for population norms, form a sufficiently homogeneous phenotype for genomic studies. The use of domain-specific ratings, which may have greater test-retest reliability and are finer-grained than global composite measures of neurocognitive function, may be less subject to noise in the detection of genetic markers [31•, 139]. The predictive value of combinations of the most promising genetic biomarkers might be evaluated in longitudinal studies that incorporate consistent case definitions, corrections for practice effects, and the best available population norms. Finally, additional studies focused on milder forms of HAND, including ANI, in individuals with undetectable virus and which use consistent diagnostic criteria are essential [140, 141]. A recent substudy of the CHARTER cohort addressed the prognostic relevance of ANI, which has been much debated, confirming that subjects with ANI experience significantly higher rates of decline to symptomatic HAND than those who are neurocognitively normal at baseline [142•].

The rapid growth of bioinformatics and systems biology as disciplines, and the evolution of machine learning tools, has now made it possible to emphasize identification of an increasing number of biological processes that underlie HAND [31•, 143•, 144•]. Microarray gene-expression and epigenetic studies can and should be further exploited for this purpose, with the caveat that they are best designed to provide broad brushstrokes, not detailed mechanistic understanding. For example, recent epigenetics work has revealed regulatory miRNA pathways validating general mechanisms such as global mitochondrial dysfunction. The *CREB* gene and its targets, implicated in both histone-modification and miRNA studies of HAND [105•, 114•], have complex roles in cell growth, differentiation, and neuronal function.

Conventional statistical approaches often fail to identify weak single-gene and gene-gene (or gene-environment) interaction effects, which are actually likely to be the fundamental genetic drivers of complex phenotypes like HAND [31•]. Network methods have emerged as a more powerful way to detect such effects. In order to derive clinically meaningful understanding of HAND and highlight biological processes that can be targeted therapeutically, it will be helpful to utilize the latest tools for integrating data from multiple sources and of multiple types. Several computational tools have been developed in recent years to predict the impact of a nonsynonymous genetic variants on protein function and, hence, distinguish pathogenic from neutral variants [145•]. Techniques such as WGCNA [146] hold promise for more powerful delineation of disease-related co-expression modules that account for functional relatedness of genes, and which can be used to analyze data from a large variety of “omics” venues.

The internal consistency of microarray-based transcriptomic and epigenetic studies of HAND is difficult to know, as this has not been well studied; very small numbers of subjects (<5) are often evaluated, and rarely have subjects served as their own controls. Many potential confounding and modifying factors are also in play. Given small anticipated effect sizes and the inability to estimate power in most genome-wide studies conducted for discovery purposes, it is imperative that replicative studies now be performed in larger numbers of subjects with estimates of clinically meaningful effect sizes, measurement error, and potential confounders in mind. In addition, standardization of research protocols and assays going forward may be helpful for combining data across multiple cohorts in order to meet the stringent power requirements of GWAS and also to facilitate validation of true-positive findings. Whole-exome sequencing approaches, which have not been published in HAND, are another way to identify the functional genomic variation that is responsible for common complex diseases without the high costs associated with whole-genome sequencing, while maintaining high coverage and sequence depth [147]. Research in the area will also benefit from reporting of both corrected and uncorrected *p*-values, so that type I and type II error can be balanced in the ongoing process of replication. The biology of replicated genes and pathways will then require further exploration at the bench to determine which ones are therapeutically actionable.

### Refinement of HAND subphenotypes or intermediate phenotypes

Due to the constraints of neuropsychometric tests and HAND definitions based only on these tests, alternative phenotypic measures of HAND that are less affected by confounders should be further explored, including structural and metabolic neuroimaging indices and new neuropathological correlates of HAND in the cART era, such as synaptodendritic simplification [148, 149]. Studies emerging the neuroimaging literature suggest that structural and metabolic subphenotypes of HAND may allow for more powerful analysis of genetic impacts on HAND [100, 129•, 150•, 151•]. Other noninvasive and low-cost biomarkers of NCI are also urgently needed to assist in monitoring patients over time, including in low-resource settings, because better characterization of longitudinal trajectories of neurocognitive performance will facilitate association of neurocognitive changes with host genomic factors. These biomarkers can then be applied in subpopulations with substantially different risk profiles, enhancing the generalizability and practical applicability of findings in this field. Continued refinement of practical tools for detecting mild HAND [141] may also make screening more acceptable and lower the cost of collection of much-needed longitudinal data. Tissue-based microarray studies evaluating multiple rather than single brain regions in affected individuals, with correlation of genetic, RNA, epigenetic, and protein expression data, are going to be essential to piece together complex patterns into coherent and therapeutically actionable mechanisms of pathogenesis using systems biology approaches.

## Conclusion

In conclusion, the complexity of HAND and the lessons learned from genomic studies to date suggest that increased methodologic rigor in study design and an integrated analytical approach employing tools from systems biology and machine learning are needed to significantly advance individualized care for persons with HAND. The pathways delineated by all types of genomic studies in this area will also become particularly meaningful when combined with data emerging from the NIH Brain Initiative and the Human Connectome Project, which promise to provide a comprehensive picture of human neural networks.
